# Principal Component Analysis versus Subject’s Residual Profile Analysis for Neuroinflammation Investigation in Parkinson Patients: A PET Brain Imaging Study

**DOI:** 10.3390/jimaging8030056

**Published:** 2022-02-25

**Authors:** Rostom Mabrouk

**Affiliations:** Department of Computer Science, Bishop’s University, Sherbrooke, QC J1M 1Z7, Canada; rostom.mabrouk@ubishops.ca

**Keywords:** Lrrk2, PCA, SRP, neuroinflammation, Parkinson’s disease, PBR28

## Abstract

Dysfunction of neurons in the central nervous system is the primary pathological feature of Parkinson’s disease (PD). Despite different triggering, emerging evidence indicates that neuroinflammation revealed through microglia activation is critical for PD. Moreover, recent investigations sought a potential relationship between Lrrk2 genetic mutation and microglia activation. In this paper, neuroinflammation in sporadic PD, Lrrk2-PD and unaffected Lrrk2 mutation carriers were investigated. The principal component analysis (PCA) and the subject’s residual profile (SRP) techniques were performed on multiple groups and regions of interest in 22 brain-regions. The ^11^C-PBR28 binding profiles were compared in four genotypes depending on groups, i.e., HC, sPD, Lrrk2-PD and UC, using the PCA and SPR scores. The genotype effect was found as a principal feature of group-dependent ^11^C-PBR28 binding, and preliminary evidence of a MAB-Lrrk2 mutation interaction in manifest Parkinson’s and subjects at risk was found.

## 1. Introduction

There is increasing evidence of microglia, mediators of the neuroinflammatory mechanisms, involvement in several neurodegenerative diseases [1,2,3]. Activated microglial cells have been observed in Alzheimer’s disease (AD) [4], schizophrenia [5], multiple sclerosis [6,7], Lewy body dementia (LBD) [8,9] and Parkinson’s disease (PD) [10,11].

The presence of inflammation mechanisms in PD has been demonstrated by several in vivo and post-mortem studies even though the relationship between PD pathogenesis and neuroinflammation is still uncertain [12,13]. Peripheral immune mediators and microglia activation have been observed in the substantia nigra and putamen of PD patients using immunohistochemical staining [13]. Imaging studies with ^11^C-PK11195, a first-generation translocator protein (TSPO) tracer, have shown increased microglia activation in the pons, basal ganglia and frontal and temporal cortices, suggesting an anatomically widespread distribution of microglial activation [14]. A direct link between neuroinflammation and dopaminergic degeneration has been brought forward by a recent imaging study. In that investigation, the authors found that, in the midbrain and putamen, the [^11^C]PK11195 binding potential levels were inversely correlated with [^11^C]CFT (a marker of the dopamine transporter density) binding potential values and positively correlated with the severity of motor symptoms, suggesting that neuroinflammation associated with microglial activation may indeed be part of disease progression [15].

Evidence of a selective vulnerability of the dopaminergic system to neuroinflammation is also coming from animal studies. Studies performed in rats have shown that a 2 μL intranigral injection of a solution containing 1 mg of lipopolysaccharide (LPS) per mL resulted in a significant activation of microglia. Activated microglia were accompanied by the loss of dopaminergic neurons, supporting the hypothesis that inflammation can be a trigger of the degeneration of the nigrostriatal dopaminergic system [16]. A thorough review of in vivo and in vitro studies of LPS animal models for PD is given by Liu et al. [17].

Leucine-rich repeat kinase 2 (Lrrk2) gene mutations are the most common genetic risk factor for PD [18,19,20]; recent findings showed that Lrrk2 mRNA is expressed in microglia, and the protein is expressed in all central nervous system cell types while appearing in neuronal and glial inclusions in PD patients [21]. Rodent studies also showed that LPS-activated microglial cells from transgenic mice overexpressing the Parkinson’s disease-linked Lrrk2 (R1441G) mutation exhibit increased expression and secretion of proinflammatory cytokines compared with wild-type control microglia [22]. Similar findings indicate that the presence of Lrrk2 with G2019S and R1441G mutation in small animal microglial cells sensitizes them toward a neurotoxic state role [23,24,25,26,27]. Given the prominent role of Lrrk2 in the regulation of the inflammatory process and the link between Lrrk2 mutation and PD, it is of interest to investigate if Lrrk2 mutation carriers either in the symptomatic or asymptomatic stage manifest increased neuroinflammation. A recent study found that symptomatic Lrrk2 mutation carriers showed increased levels of pro-inflammatory marker fatty-acid protein, similar to sporadic PD (sPD), but, unlike sPD, they did not show increased levels of the pro-inflammatory markers of interleukin-12-p40. Additionally, non-manifesting mutation carriers exhibited a control-like peripheral inflammatory profile [28]. While this recent study concentrated on peripheral markers, to the best of our knowledge, no reported data exist for the neuroinflammatory processes in this patient population and subjects at risk of developing PD.

In this study, we investigated if neuroinflammation is present in lrrk2 mutation carriers and we provided preliminary insights into its relation to PD development. We also explored the hypothesis that neuroinflammation precedes dopaminergic deficit. The approach followed to confirm or reject the hypothesis; the investigation of a possible presence of neuroinflammation in asymptomatic and symptomatic Lrrk2 mutation carriers was performed and compared to the outcomes of similar measurements performed on sPD subjects. The goal was two-fold. First, it was to evaluate the SUV measurement against V_T_ of ^11^C-PBR28 tracer. Second, it was to investigate the SUV profile in different groups.

While exhibiting a much greater signal to noise ratio compared to ^11^C-PK11195, binding of ^11^C-PBR28 is known to be sensitive to the rs6971 single nucleotide polymorphism in the TSPO gene [29,30,31]. According to the binding affinity phenotype, the population is classified into three groups, low-affinity binders (LAB), mixed affinity binders (MAB) and high-affinity binders (HAB). LABs are relatively rare in the general population (<10%) and were excluded from this investigation. 

In addition to the need of accounting for the differential binding according to TSPO polymorphism, the analysis of ^11^C-PBR28 data is complicated by the fact that it is not always possible to find a suitable reference region devoid of specific binding. The theoretically most appropriate analysis would thus employ methods using an arterial plasma input function to derive the tracer total distribution volume (V_T_). Nevertheless, V_T_ values derived from time–activity curves of second-generation ligands are characterized by their high inter-subject variation. To overcome this issue, investigators used the cerebellum as a pseudo-reference tissue estimate of DVR in Alzheimer study [32]. The normalization of V_T_ by the measured free fractions (*fp*) was also explored in multiple sclerosis studies [33], and the normalization of V_T_ by the whole brain V_T_ was explored in a schizophrenia study [34].

These results indicated a substantial decrease in the inter-subjects’ variability even though they still have certain limits. The use of the cerebellum as a pseudo-reference for ^11^C-PBR28 binding has been found adequate for the Alzheimer disease subjects; however, our data revealed a significant difference between the groups in this region. The plasma *fp* is very small (5–10%) and, hence, it is very sensitive to measurement error, so caution should be taken before using the whole brain activity normalization of the regional V_T_ outcomes because of the bias that could be introduced due to the small difference in the global means of the different groups.

Here, we used the standard uptake value (SUV) drawn from a set of regions (SUVs were calculated as the average of the left and the right regional side in bilateral regions) as a primary outcome to analyze our data. The SUV values were validated against V_T_ values in a subset of subjects where the cannulation was successfully performed.

In order to minimize the potential methodological confounds associated with SUV values, an analysis was performed on multiple groups and regions of interest. We applied principal component analysis (PCA) to extract differences between the groups. The scores of the retained principal components (PCs) obtained from the PCA decomposition were used to discriminate between the groups, and the correlations between the different effects (genotype, disease duration) and PCs scores were used as descriptors of the covariance pattern. We compared the ^11^C-PBR28 binding profile in four groups, i.e., HC, sPD, Lrrk2-PD and lrrk2-UC, in MAB and HAB groups using PCs scores obtained from absolute SUV and the subject’s residual profile (SPR) analysis.

## 2. Material and Methods

### 2.1. Subject and Data Acquisition

Original data included in this study were obtained from the Brain Research Centre, University of British Columbia. A total of 35 subjects were considered and consisted of healthy controls (11 subjects, 4 MABs and 7 HABs), G2019S LRRK2 mutation carriers without Parkinson’s disease (PD) symptoms (8 subjects, 5 MABs and 3 HABs) and 16 subjects diagnosed with PD (16 subjects, 7 Sporadic- and 4 LRRK2-PD in MABs and 2 sporadic- and 2 Lrrk2-PD in HABs). PET scan was completed in conjunction with MRI scan on each subject. The PET scans were performed on the Siemens High-Resolution Research Tomograph (HRRT) at the UBC PET Imaging Centre. 11C-PBR28 data were acquired for 120 min in list mode after 60 sec bolus venous injection of 700 ± 73 MBq (18.9 ± 1.9 mCi) of 11C-PBR28 and then reconstructed into a dynamic sequence of images (4 × 1 min, 3 × 2 min, 8 × 5 min, 7 × 10 min) using 3D ordinary Poisson-ordered subset expectation maximization (OP-OSEM) algorithm [35]. All PET data were corrected for dead time, attenuation, scatter, random events and normalization. Each subject also underwent a T1weighted anatomical magnetic resonance imaging (MRI) scan, performed on a 3T Philips Achieva camera (repetition time (TR) = 0.81 milliseconds, echo time (TE) = 8.1 milliseconds and the flip angle = 8). The acquisition matrix measures 256 × 256 × 170, and the FOV measures 50 cm.

### 2.2. Image Registration

Image registration was done using Statistical Parametric Mapping (SPM12; https://www.fil.ion.ucl.ac.uk/spm-statistical-parametric-mapping, accessed on 16 February 2022) software. Each MRI image was resampled, first, to match the voxel size of the PET images. The resampled imagen was then coregistered to the corresponding mean PET image using normalized mutual information to estimate the affine transformation for each subject. The resized MRI image was afterward warped into the Montreal Neurological Institute (MNI; https://neuroconductor.org/help/mni/, accessed on 16 February 2022) space and their inverse deformation fields vectors were applied to region-of-interest (ROI) templates predefined in the MNI space to bring them into the individual subject’s PET space in a single step. The quality of each processing step was visually checked for all scans.

### 2.3. Demographic and Clinical Characteristics of Subjects

One-hundred-twenty min ^11^C-PBR28 PET scans were performed on the Siemens High-Resolution Research Tomograph (HRRT) scanner and 3D PET brain images were reconstructed into a 256 × 256 × 170 matrix. Demographic and clinical characteristics of subjects under study are summarized in Table 1.

### 2.4. Kinetic Modeling

**Figure 1:**
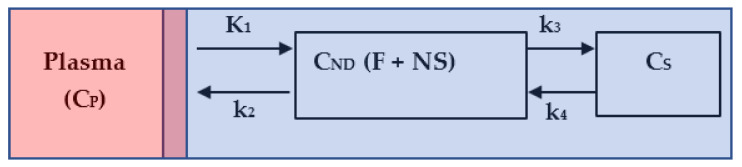
Two-tissue compartment model. The red area represents the total plasma, the mixed area represents the input to the tissue derived from the plasma, and the blue area represents the tissue: non-displaceable, that is, free plus nonspecific (F + NS), and the specific (S).

Kinetic modelling in brain PET studies is commonly used to measure a target receptor density of a particular structure in terms of specific radioligand binding potential (BP). It consists of the intravenous bolus injection of a radioligand, such as ^11^C-PBR28, and then measuring the concentration of this radioligand in plasma and in the target tissue to derive metrics that reflect receptor density. Concretely, we mean by specific BP the amount of radioligand that is associated with the target receptor, which is different from free radioligand in solution and nonspecifically associated with other macromolecular components. In some cases, and because of the complexity of the assessment of this metric (specific binding), we calculate the total volume of distribution (V_T_). V_T_ is defined as the ratio of the concentration of radioligand in a region of tissue to that in plasma. However, the tissue may contain radioligand that is specifically bound to receptors (S), nonspecifically bound (NS) or free in tissue water (F). Thus, the total concentration of radioligand in the tissue (C_PET_) can be expressed as sum of two-tissue compartment, as depicted in Figure 1.

The quantity *C_ND_* represents the concentration of the free and nonspecific binding of the radiotracer in tissue, and *C_S_* is the concentration of the specific binding of the radioligand in tissue. The quantity *C_P_* is the plasma–time–activity curve; it refers to the IF for the model. The sum of *C_ND_* and *C_S_* plus a blood volume vascular fraction ϑ(0≤ϑ<1) of $C_{p}$ produces the measured data $C_{PET}$. The differential equations describing the radioligand kinetic behavior are given by:

(1) $$\begin{array}{l} \frac{dC_{ND\;}\left(t\right)}{dt}\;=\;K_{1\;}C_{p}\left(t\right)-\left(k_{2}+k_{3}\right)C_{ND}\left(t\right)+k_{4}C_{S}\left(t\right)\\ \frac{dC_{S}\left(t\right)}{dt}=\;k_{3\;}\;C_{ND}\left(t\right)\;-\;k_{4}C_{S}\left(t\right)\\ \;\;\;\;\;\;\;C_{PET}\left(t\right)\;=\;\left(C_{ND}\left(t\right)\;+\;C_{S}\left(t\right)\right)\;+\;\vartheta C_{p}\left(t\right) \end{array}$$

where $K_{1}$ refers to the rate of delivery of the radioligand to tissue in units of volume of blood per mass of tissue per minute (mL/g/min) and $k_{2}$,$\;k_{3}$,$\;k_{4}$ are the transport rate constants in units of min^−1^. The resulting $C_{PET}\left(t\right)$ is given by:

(2) $$C_{PET}\left(t\right)\;=\;\left(\frac{K_{1}}{\alpha_{2}+\alpha_{1}}\left[\left(k_{3}+k_{4}-\alpha_{1}\right)\;\exp\left(-\alpha_{1}t\right)\;+\;\left(\alpha_{2}-k_{3}-k_{4}\right)\;\exp\left(-\alpha_{2}t\right)\right]\;⊗\;C_{p}\left(t\right)\right)\;+\;\vartheta C_{p}\left(t\right)\;$$

The *V_T_*, which represents the ratio at the equilibrium of the concentration of radioligand in tissue to that in plasma (i.e., including the specific binding, nonspecific binding and free radioligand in tissue) is defined as *V_T_* = *K*_1_*/k*_2_ × (1 + *k*_3_*/k*_4_). The independent variables *K*_1_, *k*_2_, *k*_3_ and *k*_4_ are estimated by two methods: (1) the Logan plot [36] according to the following equation:

(3) $$\frac{\int_{0}^{T}C_{PET}\left(t\right)dt}{C_{PET}\left(T\right)}=VT\;\frac{\int_{0}^{T}C_{p}\left(t\right)dt}{C_{PET}\left(t\right)}+int\;,$$

where the slope of the linear phase of plot equals the distribution volume of radiotracer. The variable “*int*” is the intercept of the plot. (2) the multilinear analysis 1 [37] according to the following equation:

(4) $$C_{PET}\;=\;-\frac{V_{T}}{b}\int_{0}^{T}C_{p}\left(t\right)dt+\;\frac{1}{b}\int_{0}^{T}C\left(t\right)dt\;,$$

with the quantities $\beta_{1}=-\frac{V_{T}}{b}$ and $\beta_{2}=\frac{1}{b}$. Hence, V_T_ = −$\beta_{1}/\beta_{2}$.

To reduce variability of V_T_ between subjects with same condition, the metric is divided by the free fraction of radioligand in the plasma (*fp*). However, and contrary to what was expected, this fraction differs from subject to another and, hence, it introduced high variability in *V_T_* outcome calculus, which is in line with studies elsewhere [38].

### 2.5. 11C-PBR28 Image Analysis

Forty-eight MRI regions of interest (ROIs) were derived from a template developed in Montreal Neurological Institute MNI space. Individual PET images were co-registered to MRI, then warped to the MNI template to calculate the transformation matrix. The inverse transformation was applied to the ROI images to fit original PET data. ROIs were placed on ^11^C-PBR28 PET data to extract the mean regional time–activity curves (TACs). Regional target TACs were fitted by the linear Logan model and the multilinear analysis model (MA1). V_T_ was calculated as a slope of the Logan fit and as the ratio of the slope and the intercept of the MA1 fit. Time-averaged activity was calculated between 60-min data scan (60–120 min post-injection) and a 30-min data scans interval (60–90, 70–100, 80–110 and 90–120-min post-injection). Time-averaged activity values were then converted to SUV outcome values by dividing measured activity by the ratio of the injected dose and subject body weight.

### 2.6. Statistical Analyses

Demographic and clinical measures were compared using factorial analysis of variance (ANOVA), with TSPO genotype and diagnosis as fixed factors. The asymmetry index (AI) metric was used to evaluate the degree of similarity between region-dependent left and right SUV values. AI is defined as (SUV_Left_side_ − SUV_Right_side_)/((1/2) × (SUV_Left_side_ + SUV_Right_side_)).

For correlative analysis, linear regression and Pearson correlation determined for SUV outcome values between V_T_ and V_T_/*fp* estimated by Logan and MA1 and between V_T_ and 30-min intervals of scans derived SUV. Bonferroni correction was applied to control for multiple group comparisons and for multiple regions comparisons. Bland–Altman (B&A) plot was used for assessing agreement between V_T_ and SUV. Two tailed Fisher’s exact test was used to determine if there was a significant association between gender and TSPO genotype. A critical *p*-value was set to 0.05.

### 2.7. Principal Component Analysis Definition and Process

Principal component analysis is a technique for reducing the dimensionality of a large dataset. It uses a vector space transform to draw new axis with high variance of synthetic data known as scores. By using mathematical projection, the original data set, which may have involved many variables, can often be interpreted in just a few variables. The projection aims to get rid of correlated and redundant features and increases the interpretability. It does so by creating new uncorrelated variables that successively maximize variance. Mathematically, we aim to find the direction with maximum spread and project the data points on that direction. Let X_(**i**, **j**)_ be the column standardized matrix of i observations (in our study, the subject’s SUV values) and j variables (in our study, the 22 regions of interest). Additionally, f_i_ and u_i_ are the direction of maximum variance and a unit vector in the direction of f_i_, respectively, with $∥u_{i}\|=1$. Let x_i_ be any point of the matrix X_(**i**, **j**)_ and $x_{i}^{\prime }$ its projection on u_i_.

(5) $$∥x_{i}^{\prime }\|\;=\;proj_{u_{i}\;}\left(x_{i}\right)=\;u_{i}^{T}\;.\;x_{i}\text{ ,}$$

where (.) is the dot product.

We have to find ui such that the $\mathrm{variance}\;\mathrm{of}\;({\mathrm{proj}}_{u_{i}\;}\left(x_{i}\right))=\mathrm{var}\left(u_{i}^{T}.x_{i}\right)$ is maximum.

(6) $$var\left(\;u_{i}^{T}.x_{i}\right)\;=\;\frac{1}{j}\;\overset{j}{\underset{1}{\sum }}{\left(u_{i}^{T}.x_{i}\right)}^{2}\;,$$

Since the matrix **X**_(**i**, **j**)_ is standardized, the optimization problem becomes:

$$max_{u}\frac{1}{j}\;\sum_{1}^{j}{\left(u_{i}^{T}.x_{i}\right)}^{2}\;\mathrm{subject}\;\mathrm{to}\;\;∥u_{i}\|\;=\;1.$$

Hence,

$$\frac{1}{j}\;\overset{j}{\underset{1}{\sum }}{\left(u_{i}^{T}.x_{i}\right)}^{2}=\;u^{T}\frac{\left(X^{T}X\right)}{j}u=\;u^{T}Su\;,$$

where *S* is the covariance matrix.

To solve this problem, we use the Lagrange’s multiplier, and we get the solution as:

(7) Su= λu 

where u is the eigenvector of the covariance matrix and λ is its eigenvalue. The direction with maximum spread is defined by the projection of data on the eigenvector corresponding to the largest λ value, called first principal component; the projection on the eigenvector corresponding to the second largest eigenvalue is called second principal component and so on.

The loadings measure the correlation between a component and variables to estimate the information they share. The loadings give the proportion of the variance of the variables explained by the components [39].

In our study, PCA was applied to analyze SUV data aiming to reduce the dimensionality of the dataset consisting of 22 interrelated regions (variables) while maintaining the variation present in the dataset as much as possible. The principal components (PCs) are ordered so that the first few retain most of the variation present in all original variables. PCA was applied on the absolute SUV dataset and separately on the SRP of SUV.

Two metrics, the percent contribution of each observation and the squared cosine, were used to evaluate the goodness of the projection and the ability of the PC to explain the variance within a group of observation. The percent contribution of each observation (participant subject) is defined as a ratio of the squared score of observation, i, on the component l $\left(f_{i,l}^{2}\right)$ by the eigenvalue associated with the component $\;\lambda_{l}$. The squared cosine metrics $cos_{i,l}^{2}$ is defined as the ratio of squared score factor $f_{i,l}^{2}$ by the squared distance of an observation i to the origin of the coordinate system. The correlation between independent variables (genotype and the symptom duration) and PCs scores was calculated to interpret the meaning of the retained factors. A correlation between original data and retained components scores was used to depict regional dependent patterns. All analyses were performed with SPSS 23 (SPSS Inc., Chicago, IL, USA), and the significant level was set at *p* < 0.05. XLSTAT 2014 was used to perform PCA.

## 3. Results

The factorial ANOVA showed no significant differences in age (F_(3, 29)_ = 1.68, *p* = 0.1), as well as in the ^11^C-PBR28 injected dose (F_(3, 29)_ = 0.68, *p* =0.6 0.295) or the specific activity at the time of injection (F_(3, 29)_ = 0.97, *p* = 0.4). Fisher’s exact tests showed no significant differences in the composition of gender (*p*__cutoff_ = 0.34). The *t*-test between UPDS III values in PD-MAB and PD-HAB was not significant (*p* > 0.05). 

### 3.1. Validation of Standard Uptake Value measurement

**Figure 2:**
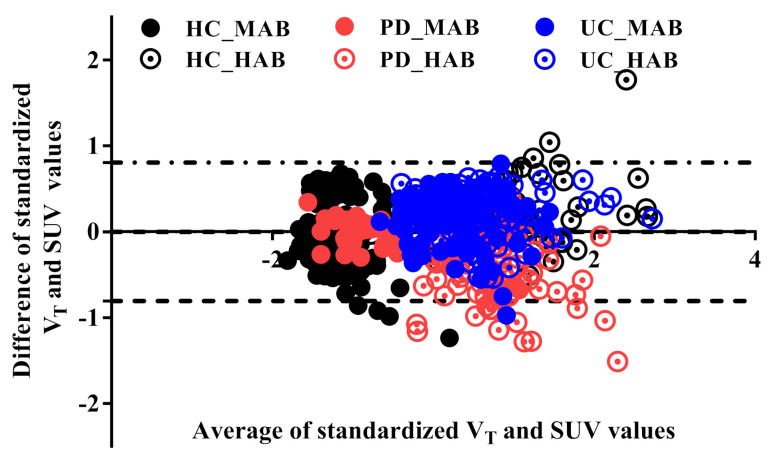
Bland–Altman plot of the standardized total distribution volume (V_T_) and the standardized SUV. The standardized Logan V_T_ is slightly higher than the standardized SUV outcome value (−0.004). The upper and lower limit agreements are −0.8 and 0.8, respectively. A few genotype- and disease-independent ROIs overflow the limit bands because of the small size of the ROIs.

A strong correlation was found between SUV_60–90_ and SUV_60–120_ (r = 0.99, *p* < 0.001), indicating that a shorter scan duration could be employed to determine this metric. SUV_60–90_ was thus used for the analysis. The one-way ANOVA showed that there were no significant differences in measured *fp* (F_(5,14)_ = 3.3, *p* = 0.8) between the three groups (HC, PD and lrrk2-UC in HAB and MAB) (Figure S1 in Supplementary Materials). Using Logan analysis, a strong correlation between SUV_60–90_ and V_T_ (R^2^ = 0.83, *p* < 0.001) and a moderate correlation between SUV_60–90_ and V_T_/*fp* were found (R^2^ = 0.5, *p* < 0.001). Using MA1, a strong correlation between SUV SUV_60–90_ and V_T_ (R^2^ = 0.77, *p* < 0.001) and a moderate correlation between SUV_60–90_ and V_T_/*fp* (R^2^ = 0.45, *p* < 0.001) were found (Figure S2 in the Supplementary Materials). The B&A plot between the standardized (Z-scores) SUV values and the standardized Logan V_T_ values (see Figure 2) revealed a very small bias (−0.04341) and 95% limits of agreement from −0.8 to 0.8. Notably, some values overflow the 95% limits agreement, which corresponds to small-sized ROIs (number of pixels ~= 300 pixels).

### 3.2. Standard Uptake Value Profile Analysis

The SUV values were highly significantly elevated in every region in HC-HAB compared to HC-MAB. Importantly, ten regions were highly significantly elevated in PD-HAB compared to PD-MAB and twelve regions highly significantly elevated in lrrk2-UC-HAB compared to lrrk2-UC-MAB (Table 2). All the regions survive the Bonferroni adjustment in HC but not in PD and lrrk2-UC.

In the MAB groups, post hoc Bonferroni tests showed that the anterior cingulate, dorsolateral prefrontal cortex (DLPFC), orbitofrontal cortex (OFC), parietal and ventricular system (VS) regions had significantly higher SUV in PD than in HC, and the anterior cingulate, anterior frontal, DLPFC, dentate nucleus, hypothalamus, medulla, OFC, posterior cingulate, parietal, pons, temporal and cerebellum regions had significantly higher SUV in lrrk2-UC than in HC. These significant regions did not survive to the multiple region correction. In the HAB groups, all the comparisons were not significant.

### 3.3. Principal Component Analysis

(1) Projection of absolute SUV on principal components

**Figure 3:**
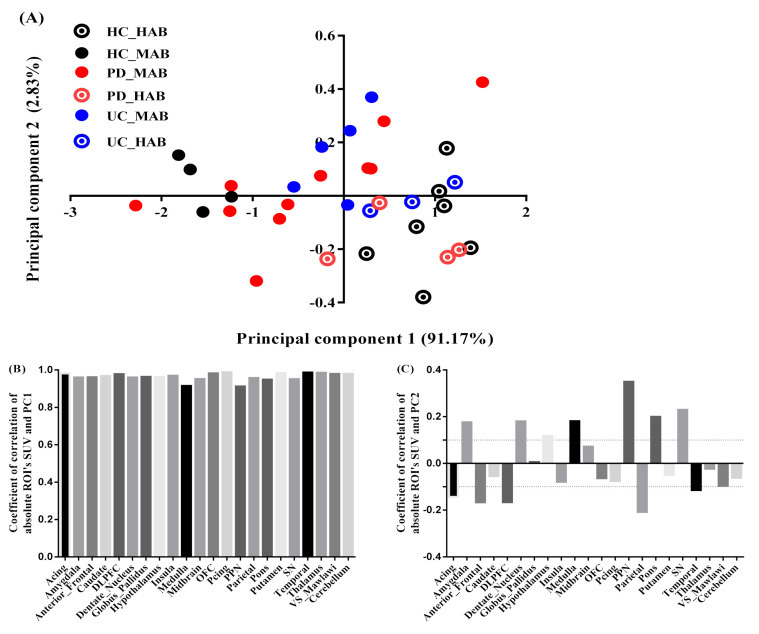
PCA absolute SUV characteristics. (**A**) Factor scores of the observations plotted on the first two components. PC1 explained 91% of the variance and PC2 explained 3% of the variance. (**B**) Coefficients of correlation between ROI’s SUV and PC1. (**C**) Coefficients of correlation between ROI’s SUV and PC2.

The two most significant axes were retained for the exploration of absolute SUV. The variance explained by the first and second principal component was equal to 94% of the total variance (Figure 3A), with the PC1 axis accounting for 91% and PC2 for only 3% (quality and projection goodness are shown in Table S6 in the Supplementary Materials). 

The regional SUV values were strongly correlated with the PC1 scores for all the brain regions (Figure 3B). PC2, however, was correlated (correlation above 0.1 is deemed important) positively with seven regions: pedunculopontine nucleus (PPN) (r = 0.32, contribution of the variable = 18%), medulla (r = 0.25, contribution of the variable = 13%), substantia nigra (SN) (r = 0.21, contribution of the variable = 9%), pons (r = 0.19, contribution of the variable = 6%), amygdala (r = 0.17, contribution of the variable = 3%), dentate nucleus (r = 0.15, contribution of the variable = 3%) and hypothalamus (r = 0.13, contribution of the variable = 3%), and correlated negatively with 5 regions: parietal (r = −0.24, contribution of the variable = 7%), dorsolateral prefrontal cortex (DLPFC) (r = −0.22, contribution of the variable = 7.7%), anterior frontal (r = −0.2, contribution of the variable = 6%), anterior cingulate (r =0.16, contribution of the variable = 4%) and temporal (r = −0.15, contribution of the variable = 2.6%) (Figure 3C).

(2) Projection of the subject’s residual profile on principal components

Similarly, the two most significant axes were retained for the exploration of the regional residual SUV profile. The variance explained by the first and second component is equal to 50% of the total variance, with the PC1 axis accounting for 29% and PC2 accounting for 20% (quality and projection goodness are shown in Table S7 in the Supplementary Materials). 

**Figure 4:**
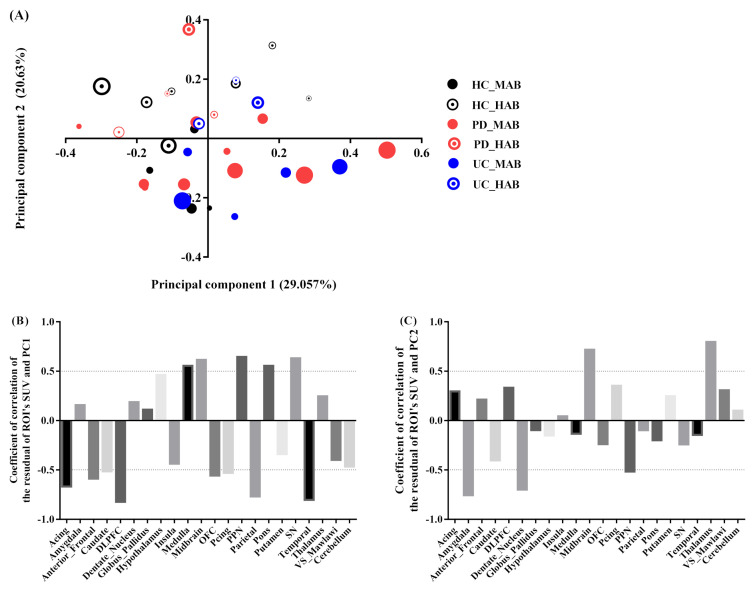
PCA subject’s residual SUV characteristics. (**A**) Factor scores of the observations plotted on the first two components. PC1 explained 29% of the variance and PC2 explained 21% of the variance. The size of markers increases with age. (**B**) Coefficients of correlation between subject’s residual profile of SUV and PC1. (**C**) Coefficients of correlation between subject’s residual profile of SUV and PC2.

The PC1 scores had a significant positive correlation with SRP in the midbrain (r = 0.61, contribution of the variable = 17%), pons (r = 0.55, contribution of the variable = 5.4%), SN (r = 0.63, contribution of the variable = 7.7%) and PPN regions (r = 0.64 contribution of the variable = 11.5%), and a significant negative correlation with SRP in the temporal (r = −0.80, contribution of the variable = 3.8%), parietal (r = −0.76, contribution of the variable = 9.6%), anterior cingulate (r = −0.66, contribution of the variable = 3%), anterior-frontal (r = −0.58, contribution of the variable = 3%) and DLPFC regions (r = −0.82, contribution of the variable = 6%). (Figure 4B). The PC2 scores were positively correlated with RSP in the thalamus (r = 0.79, contribution of the variable = 23%), midbrain (r = 0.71, contribution of the variable = 33%), and negatively correlated with the amygdala (r = −0.75, contribution of the variable = 10.6%) and dentate nucleus (r = −0.69, contribution of the variable = 8.7%) (Figure 4C).

(3) Effect of genotype on ^11^C-PBR28 SUV

**Figure 5:**
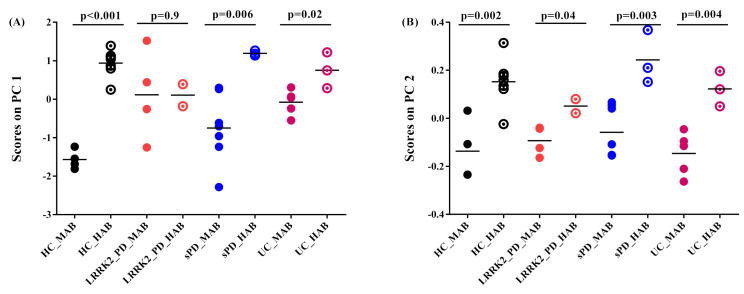
Graphs of scores on principal components. (**A**) Scores on PC1 of absolute SUV PCA decomposition. Healthy control with high-affinity binder (HC-HAB) group was significantly higher than the healthy control with mixed affinity binder (HC-MAB). Sporadic Parkinson’s disease with high-affinity binder (sPD-HAB) has a distinct trend increase toward significance compared to sporadic Parkinson’s disease with mixed affinity binder (sPD-MAB) group. lrrk2 Parkinson’s disease with high-affinity binder (lrrk2-PD-HAB) has similar scores as for Lrrk2 Parkinson’s disease with mixed affinity binder (Lrrk2-PD-MAB). Unaffected Lrkk2 mutation carriers with high-affinity binder (lrrk2-UC-HAB) group was significantly higher than unaffected Lrrk2 mutation carriers with mixed affinity binder (lrrk2-UC-MAB) group. (**B**) Scores on PC2 of PCA decomposition of SRP SUV. HC-, PD- and lrrk2-UC-HAB groups were significantly higher than HC-, sPD-, Lrrk2-PD- and lrrk2-UC-MAB matched groups.

The genotype effect was found as a principal feature of group-dependent ^11^C-PBR28 binding. This effect is observable on absolute SUV as well as in SRP-SUV analyses. The PC1 of the absolute SUV analysis shows highly significantly elevated scores in HAB-HC compared to MAB-HC (*p* = 0.001) and in HAB-sPD compared to MAB-sPD (*p* = 0.006). Moderately significantly elevated scores in HAB-lrrk2-UC compared to MAB-lrrk2-UC (*p* = 0.02) were found. The PC1 scores, however, were found obscuring the difference between HAB and MAB subjects in Lrrk2-PD (*p* = 0.9) (Figure 5A). In comparison, PC2 scores resulting from SRP SUV analysis show a significant difference between the HAB and MAB subjects in each group (in HC, *p* = 0.002; in sPD, *p* = 0.003; in lrrk2-PD, *p* = 0.04 and in lrrk2-UC *p* = 0.004) (Figure 5B).

(4) Effect of disease on ^11^C-PBR28 SUV

**Figure 6:**
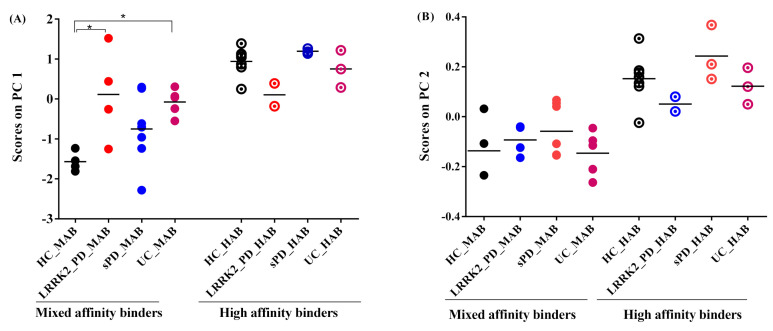
Graphs of scores on principal components. (**A**) Scores on PC1 of absolute SUV PCA decomposition. In mixed affinity binder (MAB) groups: PD and UC groups were not significantly different, whilst PD and HC were at the limit of significance and the UC group was significantly higher than HC. In high-affinity binder (HAB), however, the three groups were not significantly different from HC. (**B**) Scores on PC2 of SRP-SUV PCA decomposition. The three groups were not significantly different from HC in MAB and HAB groups. The asterisks in the plot identify significant differences between groups (*p* < 0.05).

The PC1 scores obtained from absolute SUV and PC2 scores obtained from SRP-SUV analyses stratified according to genotype illustrated the disease effect on different groups. Using one-way ANOVA followed by post hoc test, PC1 scores showed a significant difference between HC and lrrk2-UC (*p* = 0.04) and a significant difference between HC and Lrrk2-PD (*p* = 0.03) in MAB groups. No significant differences between sPD and Lrrk2-PD, between Lrrk2-PD and lrrk2-UC and between sPD and lrrk2-UC were found (*p* = 0.5, *p* = 0.4 and *p* = 0.9, respectively). In the HAB groups, only a significant difference in scores was found between Lrrk2-PD and sPD (Figure 6A).

With a one-way ANOVA followed by post hoc test, the PC2 scores of the SRP SUV showed no significant difference between groups in MABs and HABs (Figure 6B).

(5) Correlation between ^11^C-PBR28 SUV and symptom duration

**Figure 7:**
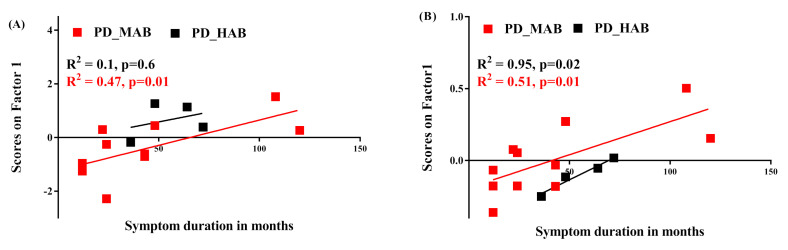
Correlation between symptom duration and principal components. (**A**) PC1 of absolute SUV PCA decomposition positively correlates with Parkinson’s disease patients with mixed affinity binder (PD-MAB). (**B**) PC1 of SRP-SUV PCA decomposition significantly correlates with PD-MAB and with HC-HAB (R^2^ = 0.51, *p* = 0.01 and R^2^ = 0.95, *p* = 0.02, respectively).

A significant positive correlation was observed between the subject scores on PC1 and symptoms duration in MAB-PD but not in HAB-PD (Figure 7A) when using absolute SUV analysis. However, a positive correlation between subject scores and symptom duration was observed in MAB- and HAB-PD when using the SRP SUV analysis (Figure 7B).

## 4. Discussion

Although it is now well established that chronic inflammation is a prominent feature of several neurodegenerative disorders, including PD, the contribution of lrrk2 mutation in terms of neuroinflammation manifestation is still unclear. However, for lrrk2-UC and lrrk2-linked PD, no evidence of genetic mutation has been established yet. The brain structures susceptible to be affected by this genetic mutation have been observed, whilst its consequences are still unclear. In this study, we investigated the use of 11C-PBR28 SUV to measure neuroinflammation in sPD and Lrrk2-PD patients and lrrk2-UC subjects. We defined in our study a set of 22 brain structures to have a comprehensive idea of the microglia interaction with the disease or genetic mutation presence. 

Firstly, we examined the sensitivity of SUV to the time-window length and the relationship between SUV and V_T_ to characterize the brain uptake of ^11^C-PBR28. We compared the SUVs calculated from 60 min intervals (60–120 min post-injection) to sequential 30 min intervals (60–90, 70–100, 80–110 and 90–120, respectively) -derived SUVs. We found that the SUVs calculated from the time interval 60–90 min showed a very strong correlation with SUVs calculated from the 60 min interval (R^2^ = 0.99, *p* < 0.001). Similar correlations were found using the other 30-min time intervals, which suggested that the scan time could be reduced from 120 to 90. The V_T_ values were derived from the Logan analysis and MA1 using the arterial plasma input function. The one-way ANOVA showed that there were no significant differences in the measured *fp* (F_(5,14)_ = 3.3, *p* = 0.8) or in the area under the curve of the input function (F_(5,8)_ = 2.34, *p* = 0.4). Despite the small subject size, we found a strong correlation between the SUV and Logan V_T_ (R^2^ =0.83). However, a worse correlation was found when we divided V_T_ by *fp*., likely because of the measurement error. The estimated Logan V_T_ values from the selected ROIs in addition to the whole gray matter and the whole white matter were consistent with the literature (Tables S1–S5 in the Supplementary Materials) [32,38]. Moreover, the inter-subject variability in the whole grey matter region (restricted to MAB healthy controls, *n* = 3) was lower for SUV (10%) than Logan V_T_ (16%). 

Despite the known dopamine deficit asymmetry in PD patients, we did not find any asymmetry in the SUV across ROIs (Figure S3 in the Supplementary Materials), and this finding is consistent with the ^11^C-DPA713 PET data analysis [40]. Accordingly, the SUV in each region used in the statistical analysis was calculated as the average of the left and the right measurement to minimize the noise effect.

Due to the large number of investigated brain structures in this study, the statistical analysis of the large number of ROIs has come to be associated with the decrease in statistical power because of the multiple comparison. As seen in Table 2, statistical significance disappears after correcting for multiple regions in the patient groups.

The PCA, known to be highly suitable for dimension reduction, was able to reduce the data dimension while keeping most of the information (94% and 50% of the information was kept on the first two axes of the absolute and SRP SUV profiles, respectively). Moreover, by investigating the absolute SUV and the residual profile, we were able to investigate the magnitude effect on the data.

The scores on the PC1 of the absolute SUV decomposition are strongly correlated with all the original SUV variables (0.89 < r < 0.98). No region’s pattern associated with the PC1 was observed, but this component significantly discriminates MAB from HAB in HC, sPD and barely in the lrrk2-UC groups. Whilst the scores were not able to discriminate between the MABs and HABs in the Lrrk2-PD groups (Figure 6A), the PC2 scores were very weakly correlated to the ROIs SUV and did not reveal any significant interpretation. One reason for this result might be because of the attached noise, whether as a result of a variety of conditions that can act or due to the co-registration step. Notably, the co-registration of MRI to PET did not ensure spatial consistency between given points in all the images, especially matching anatomical points to allow an accurate extraction of the tissue time–activity curves. These image flaws and artifact pitfalls led to a remarkable increase in the noise ratio in the image and, hence, reduced the efficiency of the statistical analysis.

These findings suggest that the first component explains the variation due to the difference in the magnitude of the SUV between groups and the second component explains the remaining variation within each group due to the slight difference in the regional tracer’s uptake. At a low threshold of r > 0.1 and r < −0.1, the brainstem and subcortical regions had a higher magnitude of SUV compared to the cortical regions. 

The PCA decomposition of the ROI’s residual SUV profile resulted in two main factors accounting for 50% of the total variance distributed almost equally on PC1 and PC2. PC1 accounted for 29% of the variance. The region pattern associated with this component is somewhat similar to that observed from the analysis of the absolute SUVs. Moreover, this component was found correlated to age in several groups (Figure 4), suggesting a potential age effect on the SUV profile in specific regions. PC2 accounting for 20% explained the genotype effect where it appears consistently in the four groups after taking out the magnitude information from the data in the analysis (Figure 6B). This finding suggests that the substantial covariance in the regional residual profile is mainly due to the genotype. The comparison between groups with the same genotype (Figure 6) revealed a significant difference between HC and Lrrk2-PD and between HC and lrrk2-UC in MAB. However, no difference was found between sPD and HC. 

It is also noteworthy that the SRP-PCA is of great importance as it emphasizes the heterogeneity of tracer uptake in different regions, and it is dependent on age. The general tendency of ^11^C-PBR28 binding shows a higher tracer binding in the brainstem regions (pons, midbrain and medulla) compared to the cortical regions (frontal, temporal and parietal cortices). 

These findings are in good agreement with those found elsewhere. Ouchi, Y conducted a PET study with [(11)C]DPA713 on small animals [14] and human brains [15] where they demonstrated that neuroinflammatory responses by intrinsic microglia contribute significantly to the progressive degeneration process of PD. In both studies (small animal and human), they found that the levels of [(11)C](R)-PK11195 binding potential values (a metric correlated to SUV) in the midbrain were significantly higher in PD than those in age-matched healthy subjects. Moreover, the binding potential in the midbrain was correlated inversely with [(11)C]CFT binding potential (a radiotracer that binds to the dopamine transporter) in the putamen and midbrain and correlated positively with the motor severity of parkinsonism.

Gerhard A et al. conducted PET study with [11C](R)-PK11195 and [18F]-dopa PET where they compared PD to normal controls [40]. The PD patients showed significantly increased mean levels of [11C](R)-PK11195 binding in the pons, basal ganglia and frontal and temporal cortical. Their findings confirm that widespread microglial activation is associated with the pathological process in PD.

Bartels A et al. conducted a PET study with [11C]-PK11195 using the cluster analysis [41]. The PD patients showed higher contralateral putamen BP and midbrain BP compared to the controls, although considerable overlap was seen and differences were not statistically significant. [11C]-PK11195 seems to be an unsuitable tracer for the accurate or reliable quantification of neuroinflammation.

Terada, T conducted a PET study using [11C]DPA713 to explore the effect of microglia activation in PD progression [42]. They used voxel-wise analysis and confirmed that ROI analysis showed that the quantitative values of[11C]DPA713 binding potential values were significantly higher in the brainstem in the PD group.

At last, the co-registration of MRI to PET might introduce misalignment in PET’s ROI delineation and combining the MRI and PET technologies into one scan could overcome this limitation. In this context, Carlo Cavaliere et al. summarized in Ref. [43] the current state about the potential added value of hybrid scanners for characterizing microglial and astrocytic activation, which, in turn, reduces considerably the amount of noise in images. Even then, limited studies used a hybrid scanner for the diagnosis of PD and the potential role of neuroinflammation in triggering the disease [44]. This new imaging tool offers a great opportunity to gain from the higher contrast resolution aspect provided by MR to obtain more accuracy in segmented regions directly without the need of the co-registration between the two modalities.

Our findings are in line with those quoted above regarding the high level of neuroinflammation in the brainstem. However, this concerns only established PD subjects. Notably, PET research focusing on non-affected lrrk2 mutation carriers is very scarce, and, through our study, we demonstrated that high level of activated microglia in lrrk2-UC might exist before PD manifests. These findings might improve our understanding of the Lrrk2 interaction with disease or in analyzing the data of subjects at risk of developing PD.

Other directions of analysis using different algorithms are worthy to be tested and compared to our findings to confirm or deny our results. Rundo et al., in Ref. [45], cited a state-of-the-art set of valuable analysis algorithms inspired from nature, which would be worth investigating in the future.

### Limitations

This work was a pilot study to test for the evidence of elevated brain microglial activity in sPD and Lrrk2-PD patients and unaffected Lrrk2 mutation carriers. However, the sample size is very limited considering the sensitivity of the groups to their genotype. In addition, the range of age and the number of subjects are not proportional within each genotype-dependent group.

## 5. Conclusions

Here, we compared the microglia activations in HC to those in sPD, Lrrk2-PD and unaffected Lrrk2 mutation carriers measured by PET tracer radioligands ^11^C-PBR28 and analyzed with PCA. The decomposition of the absolute SUV and the subject’s residual profile demonstrated the possibility of the presence of Lrrk2 mutation–MAB genotype interaction and a non-uniformity of ^11^C-PBR28 binding across the investigated regions of interest. This method is expected to improve our understanding of the Lrrk2 interaction with disease or in analyzing the data of subjects at risk of developing PD.

## Figures and Tables

**Table 1:** Demographic and clinical characteristics of subjects.

Subjects			Mixed Affinity Binders (MAB)				High-Affinity Binders (MAB)		
		HC	sPD	Lrrk2-PD	lrrk2-UC	HC	sPD	Lrrk2-PD	lrrk2-UC
Age		44.2 ± 16	6 ± 12	67 ± 17	51 ± 11	62 ± 16	56 ± 7	56 ± 3	53 ± 4
Gender	Male Female	4 0	4 3	3 1	3 2	3 4	3 0	0 2	1 2
UPDRS		3 ± 2	23 ± 8	24 ± 6	1 ± 2	4 ± 1	24 ± 5	25 ± 7	0.6 ± 1
DD		NA	3 ± 3	4 ± 3	NA	NA	4 ± 0.5	4.5 ± 2	NA
Tracer dose parameters	AI SA IM	743 ± 0.7 93 ± 53 3.62 ± 0.9	623 ± 76 179 ± 128 1.22 ± 0.5	634 ± 82 172 ± 111 1.32 ± 0.8	696 ± 104 158 ± 125 1.3 ± 0.6	667 ± 80 215 ± 130 1.25 ± 0.6	701 ± 62 149 ± 137 3 ± 1.9	712 ± 59 151 ± 140 3.2 ± 2.2	741 ± 4 91 ± 50 3.5 ± 2.6

UPDRS: Unified Parkinson’s Diseases Rating Scale; DD: disease duration; Inj; amount injected (MBq); SA: specific activity (GBq/µmol); IM: injected mass (µg). NA: not available.

**Table 2:** Statistics summary.

Brain Regions	Test between MAB and HAB Groups Using *t*-Test			Test Corrected for Multiple Regions Comparison Using Bonferroni Adjustment			Test between HC, PD and lrrk2-UC in MAB Groups Using ANOVA Followed by Post Hoc Test		Test Corrected for Multiple Regions Comparison Using Bonferroni Adjustment	
	HC	PD	UC	HC	PD	UC	HC/PD	HC/UC	HC/PD	HC/UC
ROI	*p values*									
Acing	**<0.001**	**0.03**	**0.02**	**0.00001**	0.09	0.06	**0.02**	**0.01**	0.15	0.07
Amygdala	**<0.001**	0.36	0.05	**0.00019**	0.39	0.07	0.13	0.1	0.18	0.11
Anterior_Frontal	**<0.001**	**0.01**	**0.02**	**0.00007**	0.09	0.06	0.07	**0.02**	0.15	0.07
Caudate	**<0.001**	**0.04**	0.05	**0.00008**	0.09	0.07	0.13	0.07	0.18	0.09
DLPFC	**<0.001**	**0.01**	**0.01**	**0.00002**	0.09	0.05	**0.04**	**0.03**	0.15	0.07
Dentate_Nucleus	**<0.001**	0.15	0.24	**0.00001**	0.20	0.25	0.12	**0.03**	0.18	0.07
Globus_Pallidus	**<0.001**	0.21	0.09	**0.00001**	0.24	0.11	0.05	0.07	0.15	0.09
Hypothalamus	**<0.001**	0.17	0.09	**0.00014**	0.21	0.11	0.09	**0.03**	0.15	0.07
Insula	**<0.001**	**0.03**	**0.04**	**0.00007**	0.09	0.07	0.22	0.06	0.25	0.09
Medulla	**<0.001**	0.17	0.96	**0.00034**	0.21	0.96	0.51	**0.01**	0.51	0.07
Midbrain	**<0.001**	0.14	**0.02**	**0.00046**	0.20	0.06	0.34	0.32	0.37	0.32
OFC	**<0.001**	0.09	**0.04**	**0.00001**	0.15	0.07	**0.04**	**0.04**	0.15	0.07
Pcing	**<0.001**	**0.04**	**0.02**	**0.00001**	0.09	0.06	0.08	**0.04**	0.15	0.07
PPN	**<0.001**	0.51	0.10	**0.00004**	0.51	0.12	0.51	0.21	0.51	0.21
Parietal	**<0.001**	**0.02**	**0.03**	**0.00001**	0.09	0.06	**0.02**	**0.006**	0.15	0.07
Pons	**<0.001**	0.12	0.10	**0.00034**	0.19	0.12	0.16	**0.03**	0.21	0.07
Putamen	**<0.001**	**0.04**	**0.004**	**0.00002**	0.09	0.05	0.08	0.07	0.15	0.09
SN	**<0.001**	0.38	0.12	**0.00032**	0.40	0.13	0.21	0.09	0.25	0.10
Temporal	**<0.001**	0.05	**0.01**	**0.00001**	0.09	0.06	0.06	**0.03**	0.15	0.07
Thalamus	**<0.001**	**0.04**	**0.03**	**0.00001**	0.09	0.06	0.09	0.07	0.15	0.09
VS	**<0.001**	0.06	**0.0006**	**0.00001**	0.09	0.004	**0.04**	0.05	0.15	0.08
Cerebellum	**<0.001**	**0.02**	0.05	**0.00004**	0.09	0.07	0.05	**0.02**	0.15	0.07

Values in bold are significant at *p* < 0.005.

## Data Availability

The data presented in this analysis are derived from collected data from Brain Research Center, University of British Columbia between 2015 and 2016. Data sheet is available on reasonable request from the corresponding author.

## Supplementary Materials

The following supporting information can be downloaded at: https://www.mdpi.com/2313-433X/8/3/56/s1, Figure S1: Free fraction measurement. The factorial ANOVA showed that there were no significant differences in measured fp (F(5,14) = 3.3, *p* = 0.8); Figure S2: Linear regression analysis between 11C-PBR28 SUV calculated from the time interval 60–90 min post-injection and VT derived with Logan plot and MA1 for the combined ROIs. Best correlation was found between SUV and Logan VT; Figure S3: Percentage change between left and right side 11C-PBR28 SUV. Small ROIs had slightly higher percentage change compared to large ROIs; Table S1: Regional 11C-PBR28 VT estimated with Logan plot in healthy control, PD and asymptomatic LRRK2 mutation carriers, stratified by genotype; Table S2: Regional 11C-PBR28 VT/fp estimated with Logan plot in healthy control, PD and asymptomatic LRRK2 mutation carriers, stratified by genotype; Table S3: Regional 11C-PBR28 VT estimated with MA1 plot in healthy control, PD and asymptomatic LRRK2 mutation carriers, stratified by genotype; Table S4: Regional 11C-PBR28 VT/fp estimated with MA1 plot in healthy control, PD and asymptomatic LRRK2 mutation carriers, stratified by genotype; Table S5: Regional 11C-PBR28 VT/fp estimated with Logan plot in healthy control, PD and asymptomatic LRRK2 mutation carriers, stratified by genotype; Table S6: Genotype and age of the raw data, coordinate, contribution of the observations to the components squared cosine of the observation for the absolute SUV PCA decomposition; Table S7: Genotype and age of the raw data, coordinate, contribution of the observations to the components squared cosine of the observation for the SUV SRP-PCA decomposition.
