# Supplementary material for: Principal Component Analysis versus Subject’s Residual Profile Analysis for Neuroinflammation Investigation in Parkinson Patients: A PET Brain Imaging Study
Source: J Imaging. 2022 Feb 25;8(3):56. doi: 10.3390/jimaging8030056 (PMC8954189; doi:10.3390/jimaging8030056)
Supplement: Supplementary file 1 [file jimaging-08-00056-s001.zip › jimaging-1555350-supplementary.pdf]

Journal of
Imaging

MDPI

Supplementary

Principal Component Analysis versus Subject's Residual Profile analysis for Neuroinflammation investigation in Parkinson Patients: A PET brain imaging study

Rostom Mabrouk

Department of Computer Science, Bishop's University, Sherbrooke, QC J1M 1Z7, Canada; rostom.mabrouk@ubishops.ca

Supplementary material

- *Measured free fraction*

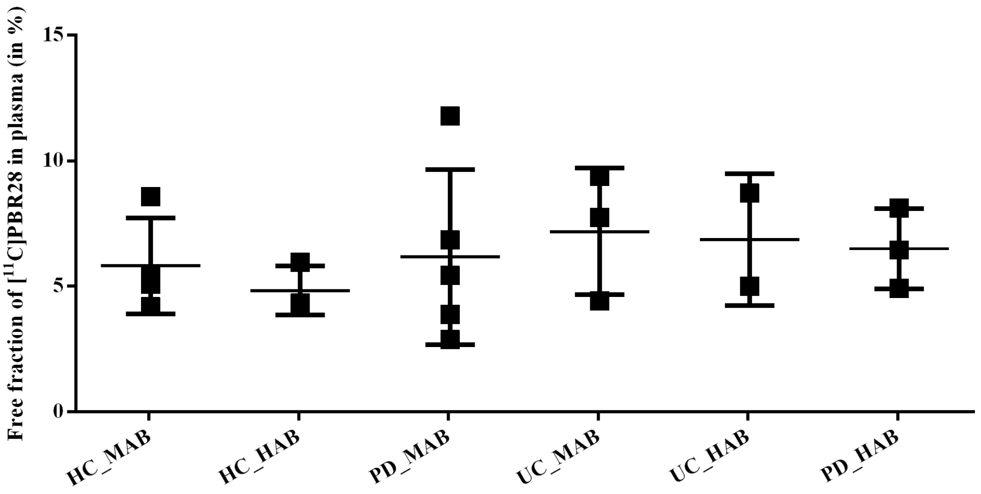

Figure S1. Free fraction measurement. The factorial ANOVA showed that there were no significant differences in measured *fp* ($F_{(5,14)}$=3.3, p=0.8).

- *Linear correlation between VT and SUV and between VT/fp and SUV*

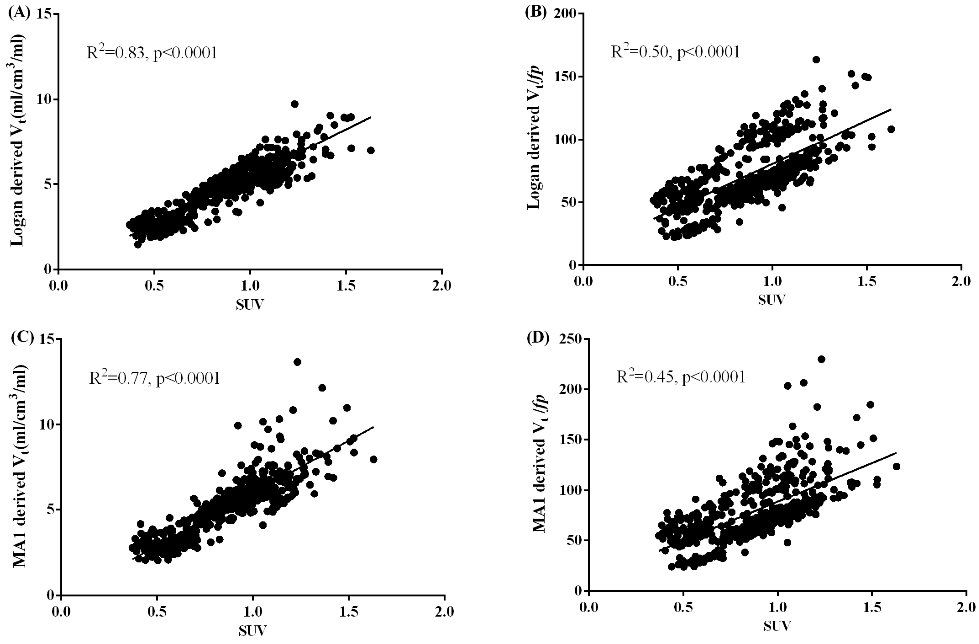

Figure S2. Linear regression analysis between ^{11}C-PBR28 SUV calculated from the time interval 60-90 minutes post-injection and V_T derived with Logan plot and MA1 for the combined ROIs. Best correlation was found between SUV and Logan V_T.

- *Asymmetry index of SUV ^{11}C-PBR28*

The asymmetry of ^{11}C-PBR28 SUV in PD was examined using the percentage change between left and right-side tracer's uptake. Fig. S1 shows small change in ROIs (% change < 10%).

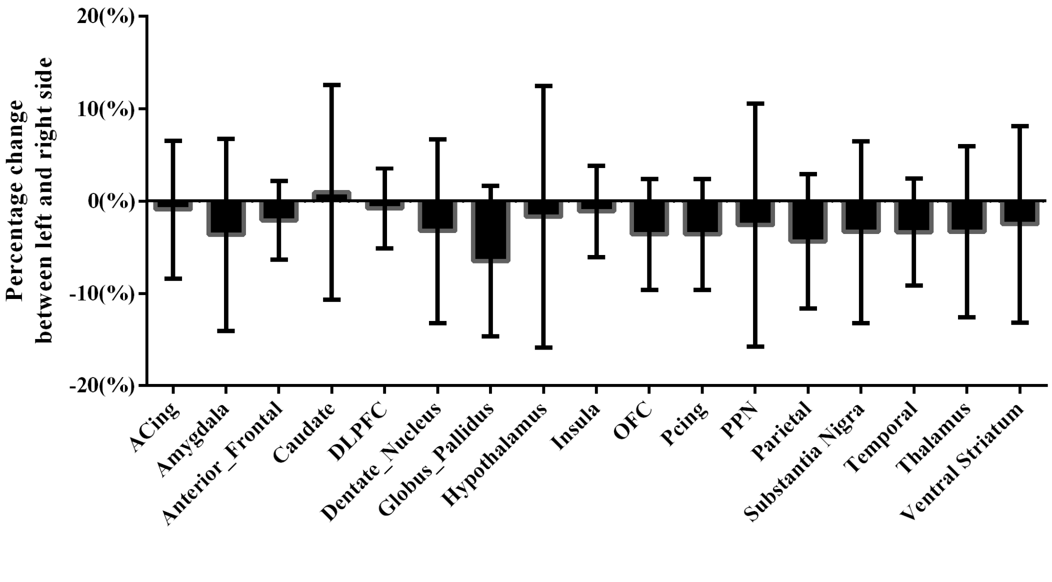

Figure S3. Percentage change between left and right side ^{11}C-PBR28 SUV. Small ROIs had slightly higher percentage change compared to large ROIs.

- *Logan plot and MA1 derived total distribution volume and standard uptake value*

Total distribution volume (V_T) derived from Logan analysis and MA1 using arterial input function. All value are presented in mean ±standard deviation except for high affinity genotyped healthy control (HAB-HC) (number of subject =1).

Table S1. Regional ^{11}C-PBR28 V_T estimated with Logan plot in healthy control, PD and asymptomatic LRRK2 mutation carriers, stratified by genotype.

		**WGM**	**WWM**	**Putamen**	**Caudate**	**SN**	**Midbrain**	**Medulla**	**Cerebellum**
**HC**	**MAB**	2.5±0.4	2.4±0.4	2.5±0.5	2.3±0.3	2.8±0.5	3.7±0.2	2.1±0.5	2.4±0.3
	**HAB**	5.5	5.3	6.7	5	8.1	8.9	6.2	5.7
**PD**	**MAB**	2.7±0.2	2.8±0.2	3.2±0.2	2.6±0.1	3.5±0.1	4.1±0.4	3.6±0.4	2.9±0.1
	**HAB**	5,5±1.1	5.2±0.7	6.1±0.6	5.1±0.8	6.±0.8	8.1±1.1	6.2±0.1	5.9±1
**UC**	**MAB**	4.5±0.3	4.4±0.0	4.6±0.3	4.±0.2	5.3±0.1	6.2±0.1	6.7±0.1	4.3±0.2
	**HAB**	5.1±0.5	4.4±0.5	5.5±0.1	4.5±0.1	6.±0.3	7.9±0.9	5.3±0.6	4.9±0.6

HC= healthy controls; PD = Parkinson's disease; UC = Asymptomatic LRRK2 mutation carriers; SN = Substantia nigra; WGM = whole gray matter; WWM = whole white matter

Table S2. Regional ^{11}C-PBR28 V_T/fp estimated with Logan plot in healthy control, PD and asymptomatic LRRK2 mutation carriers, stratified by genotype.

		WGM	WWM	Putamen	Caudate	SN	Midbrain	Medulla	Cerebellum
HC	MAB	42±16	41±16	43±11	39±12	47±16	62±13	44±13	40±12
	HAB	92	90	113	84.2	136	150	105	95
PD	MAB	40±26	40±24	44±14	36±13	51±21	55±17	53±25	43±19
	HAB	74±4	71±1	81±3	66±6	78±6	105±8	80±12	78±5
UC	MAB	53±3	52±7	55±3	47±6	63±7	73±11	79±11	51±4
	HAB	78±22	73±20	87±21	71±17	93±20	121±19	80±12	75±11

HC= healthy controls; PD = Parkinson's disease; UC = Asymptomatic LRRK2 mutation carriers; SN = Substantia nigra; WGM = whole gray matter; WWM = whole white matter

Table S3. Regional ^{11}C-PBR28 V_T estimated with MA1 plot in healthy control, PD and asymptomatic LRRK2 mutation carriers, stratified by genotype.

		**WGM**	**WWM**	**Putamen**	**Caudate**	**SN**	**Midbrain**	**Medulla**	**Cerebellem**
**HC**	**MAB**	2.5±0.3	2.4±0.4	2.8±0.2	2.7±0.4	3.1±0.5	4.1±0.1	3.1±0.4	2.5±0.3
	**HAB**	5.5	5.4	7	5.1	9.2	10.9	7.6	5.7
**PD**	**MAB**	2.2±0.2	2.8±0.1	3.3±0.2	2.7±0.1	3.9±0.3	4.2±0.3	3.9±0.4	3.1±0.2
	**HAB**	5.4±1.1	5.2±0.7	6.3±0.5	5.1±0.8	6.5±0.4	8.9±1.1	7.1±1.1	5.8±0.8
**UC**	**MAB**	4.5±0.3	4.4±0.1	4.9±0.2	4.4±0.1	5.8±0.1	6.7±0.3	7.3±0.4	4.4±0.2
	**HAB**	5.1±0.5	4.8±0.5	5.7±0.1	4.7±0.1	7.2±0.3	8.3±0.9	6.1±0.3	4.9±0.6

HC= healthy controls; PD = Parkinson's disease; UC = Asymptomatic LRRK2 mutation carriers; SN = Substantia nigra; WGM = whole gray matter; WWM = whole white matter

Table S4. Regional ^{11}C-PBR28 V_T/fp estimated with MA1 plot in healthy control, PD and asymptomatic LRRK2 mutation carriers, stratified by genotype.

		WGM	WWM	Putamen	Caudate	SN	Midbrain	Medulla	Cerebellum
**HC**	**MAB**	43±16	42±16	47±12	46±16	52±17	68±15	55±13	42±13
	**HAB**	93	90	117	86	155	184	127	97
**PD**	**MAB**	40±26	41±24	46±16	38±14	58±27	58±19	58±28	44±20
	**HAB**	74±3	72±2	85±2	69±6	89±7	121±7	97±15	78±4
**UC**	**MAB**	53±3	52±6	57±5	52±8	68±9	79±6	85±6	51±3
	**HAB**	79±22	74±20	89±22	73±18	112±26	126±21	93±20	75±10

HC= healthy controls; PD = Parkinson's disease; UC = Asymptomatic LRRK2 mutation carriers; SN = Substantia nigra; WGM = whole gray matter; WWM = whole white matter

Table S5. Regional ^{11}C-PBR28 V_T/fp estimated with Logan plot in healthy control, PD and asymptomatic LRRK2 mutation carriers, stratified by genotype.

		WGM	WWM	Putamen	Caudate	SN	Midbrain	Medulla	Cerebellum
**HC**	**MAB**	0.48±0.05	0.47±0.04	0.5±0.05	0.43±0.06	0.61±0.04	0.81±0.14	0.64±0.11	0.45±0.04
	**HAB**	0.95	0.89	1.07	0.87	1.14	1.46	1.13	0.98
**PD**	**MAB**	0.68±0.16	0.67±0.18	0.84±0.2	0.68±0.17	1.00±0.24	1.23±0.27	1.01±0.2	0.75±0.15
	**HAB**	0.90±0.11	0.84±0.13	1.01±0.14	0.84±0.11	1.00±0.12	1.35±0.21	1.09±0.18	0.94±0.1
**UC**	**MAB**	0.77±0.02	0.76±0.02	0.8±0.04	0.67±0.03	1±0.12	1.15±0.12	1,17±0.07	0.74±0.03
	**HAB**	0.92±0.08	0.85±0.07	0.95±0.05	0.8±0.09	1.12±0.05	1.47±0.15	1.09±0.23	0.92±0.13

HC= healthy controls; PD = Parkinson's disease; UC = Asymptomatic LRRK2 mutation carriers; SN = Substantia nigra; WGM = whole gray matter; WWM = whole white matter

Quality measurement of principal component analysis

Table S6. Genotype and age of the raw data, coordinate, contribution of the observations to the components squared cosine of the observation for the absolute SUV PCA decomposition.

Observation	Genotype	Age	F1	F2	Ctr 1(%)	Ctr 2(%)	Cosine^2 1	Cosine^2 2 (10^{-3})
**HC**	MAB	24	-1.81	0.15	9.3	2.1	**0.98**	6.9309
**HC**	MAB	44	-1.55	-0.06	6.7	0.3	**0.98**	1.5148
**HC**	MAB	46	-1.23	0.00	4.3	0.0	**0.95**	0.0088
**HC**	MAB	63	-1.69	0.10	8.0	0.9	**0.98**	3.3549
**HC**	HAB	37	1.13	0.18	3.6	2.9	**0.91**	22.8041
**HC**	HAB	47	1.04	0.02	3.1	0.0	**0.90**	0.2395
**HC**	HAB	53	1.39	-0.19	5.4	3.4	**0.95**	18.7070
**HC**	HAB	70	1.10	-0.04	3.4	0.1	**0.88**	1.0357
**HC**	HAB	73	0.25	-0.22	0.2	4.3	**0.35**	265.9207
**HC**	HAB	75	0.80	-0.12	1.8	1.2	**0.86**	18.0631
**HC**	HAB	80	0.87	-0.38	2.1	13.0	**0.74**	139.9935
**PD**	MAB	42	-2.28	-0.04	14.7	0.1	**1.00**	0.2554
**PD**	MAB	45	-0.96	-0.32	2.6	9.2	**0.88**	97.3724
**PD**	MAB	48	-0.26	0.08	0.2	0.5	**0.45**	39.1425
**PD**	MAB	57	-1.25	-0.06	4.4	0.3	**0.96**	2.0437
**PD**	MAB	66	0.26	0.10	0.2	1.0	**0.55**	85.6147
**PD**	MAB	66	-0.71	-0.09	1.4	0.7	**0.91**	13.7263
**PD**	MAB	67	-0.61	-0.03	1.1	0.1	**0.88**	2.4394
**PD**	MAB	69	-1.24	0.04	4.3	0.1	**0.97**	0.8846
**PD**	MAB	75	0.30	0.10	0.2	0.9	**0.54**	63.4546
**PD**	MAB	78	0.44	0.28	0.5	7.1	**0.49**	197.9208
**PD**	MAB	86	1.52	0.43	6.5	16.4	**0.90**	70.2678
**PD**	HAB	48	1.26	-0.20	4.5	3.7	**0.95**	24.4233
**PD**	HAB	52	0.39	-0.03	0.4	0.1	**0.80**	3.7266
**PD**	HAB	56	-0.18	-0.24	0.1	5.1	0.27	**465.9003**
**PD**	HAB	62	1.14	-0.23	3.6	4.8	**0.88**	35.8303
**UC**	MAB	37	-0.24	0.18	0.2	3.0	**0.34**	190.7016
**UC**	MAB	42	0.04	-0.03	0.0	0.1	0.03	19.5675
**UC**	MAB	54	0.07	0.24	0.0	5.4	0.04	**498.8454**
**UC**	MAB	61	0.31	0.37	0.3	12.4	0.34	**495.5486**
**UC**	MAB	64	-0.55	0.03	0.8	0.1	**0.86**	3.1726
**UC**	HAB	49	0.75	-0.02	1.6	0.0	**0.81**	0.7558
**UC**	HAB	55	0.29	-0.06	0.2	0.3	**0.53**	19.8147
**UC**	HAB	67	1.22	0.05	4.2	0.2	**0.96**	1.6747

Table S7. Genotype and age of the raw data, coordinate, contribution of the observations to the components squared cosine of the observation for the SUV SRP-PCA decomposition.

Observation	Genotype	Age	F1	F2	Ctr 1(%)	Ctr 2(%)	Cosine^2 1	Cosine^2 2
**HC**	MAB	24	0.004	-0.235	0.0	6.5	0.00	**0.62**
**HC**	MAB	44	-0.164	-0.107	2.3	1.4	**0.29**	0.12
**HC**	MAB	46	-0.038	0.032	0.1	0.1	0.02	0.01
**HC**	MAB	63	-0.046	-0.236	6.8	6.6	0.02	**0.53**
**HC**	HAB	37	0.283	0.135	0.2	2.2	**0.46**	0.11
**HC**	HAB	47	0.181	0.314	2.8	11.7	0.17	**0.50**
**HC**	HAB	53	-0.102	0.159	0.9	3.0	0.13	**0.30**
**HC**	HAB	70	0.078	0.185	0.5	4.1	0.03	0.17
**HC**	HAB	73	-0.173	0.122	2.5	1.8	**0.26**	0.13
**HC**	HAB	75	-0.111	-0.024	1.0	0.1	0.16	0.01
**HC**	HAB	80	-0.299	0.176	7.5	3.7	**0.34**	0.12
**PD**	MAB	42	-0.178	-0.152	2.7	2.8	**0.48**	0.36
**PD**	MAB	45	-0.362	0.041	11.1	0.2	**0.84**	0.01
**PD**	MAB	48	0.053	-0.043	0.2	0.2	0.04	0.02
**PD**	MAB	57	-0.177	-0.164	2.7	3.2	**0.28**	0.24
**PD**	MAB	66	0.154	0.067	2.0	0.5	**0.36**	0.07
**PD**	MAB	66	-0.180	-0.154	2.7	2.8	**0.41**	0.30
**PD**	MAB	67	-0.032	0.054	0.1	0.3	0.02	0.07
**PD**	MAB	69	-0.067	-0.155	0.4	2.8	0.06	**0.33**
**PD**	MAB	75	0.076	-0.108	0.5	1.4	0.08	0.17
**PD**	MAB	78	0.271	-0.124	6.2	1.8	**0.36**	0.07
**PD**	MAB	86	0.503	-0.040	21.3	0.2	**0.73**	0.00
**PD**	HAB	48	-0.114	0.152	1.1	2.7	0.17	**0.29**
**PD**	HAB	52	0.017	0.080	0.0	0.8	0.01	0.15
**PD**	HAB	56	-0.250	0.021	5.3	0.1	**0.68**	0.01
**PD**	HAB	62	-0.054	0.368	0.2	16.0	0.01	**0.56**
**UC**	MAB	37	0.075	-0.263	0.5	8.2	0.04	**0.55**
**UC**	MAB	42	-0.057	-0.046	0.3	0.2	0.06	0.04
**UC**	MAB	54	0.218	-0.115	4.0	1.6	**0.41**	0.11
**UC**	MAB	61	0.370	-0.095	0.4	1.1	**0.72**	0.05
**UC**	MAB	64	-0.072	-0.210	11.6	5.3	0.07	**0.61**
**UC**	HAB	49	0.079	0.196	0.5	4.6	0.04	0.25
**UC**	HAB	55	-0.025	0.050	0.1	0.3	0.01	0.03
**UC**	HAB	67	1.22	0.05	4.2	0.2	**0.96**	1.6747
